# Supplementary material for: Different Periampullary Types and Subtypes Leading to Different Perioperative Outcomes of Pancreatoduodenectomy: Reality and Not a Myth; An International Multicenter Cohort Study
Source: Cancers (Basel). 2024 Feb 23;16(5):899. doi: 10.3390/cancers16050899 (PMC10930966; doi:10.3390/cancers16050899)
Supplement: Supplementary file 1 [file cancers-16-00899-s001.zip › cancers-2795478-supplementary.pdf]

Different Periampullary Types and Subtypes Leading to Different Perioperative Outcomes of Pancreatoduodenectomy: Reality and Not a Myth; An International Multicenter Cohort Study

SUPPLEMENTARY FILE

Bas A. Uijterwijk [1,2,3], Daniël H. Lemmers [1,2,3], Giuseppe Kito Fusai [4], Bas Groot Koerkamp [5], Sharnice Koek [6], Alessandro Zerbi [7], Ernesto Sparrelid [8], Ugo Boggi [9], Misha Luyer [10], Benedetto Ielpo [11], Roberto Salvia [12], Brian K. P. Goh [13,14], Geert Kazemier [15], Bergthor Björnsson [16], Mario Serradilla-Martín [17], Michele Mazzola [18], Vasileios K. Mavroeidis [19,20], Santiago Sánchez-Cabús [21], Patrick Pessaux [22], Steven White [23], Adnan Alseidi [24], Raffaele Dalla Valle [25], Dimitris Korkolis [26], Louisa R. Bolm [27], Zahir Soonawalla [20], Keith J. Roberts [28], Miljana Vladimirov [29], Alessandro Mazzotta [30], Jorg Kleeff [31], Miguel Angel Suarez Muñoz [32], Marc G. Besselink [2] and Mohammed Abu Hilal [1,*] on behalf of ISGACA Consortium

Supplementary figure S1:

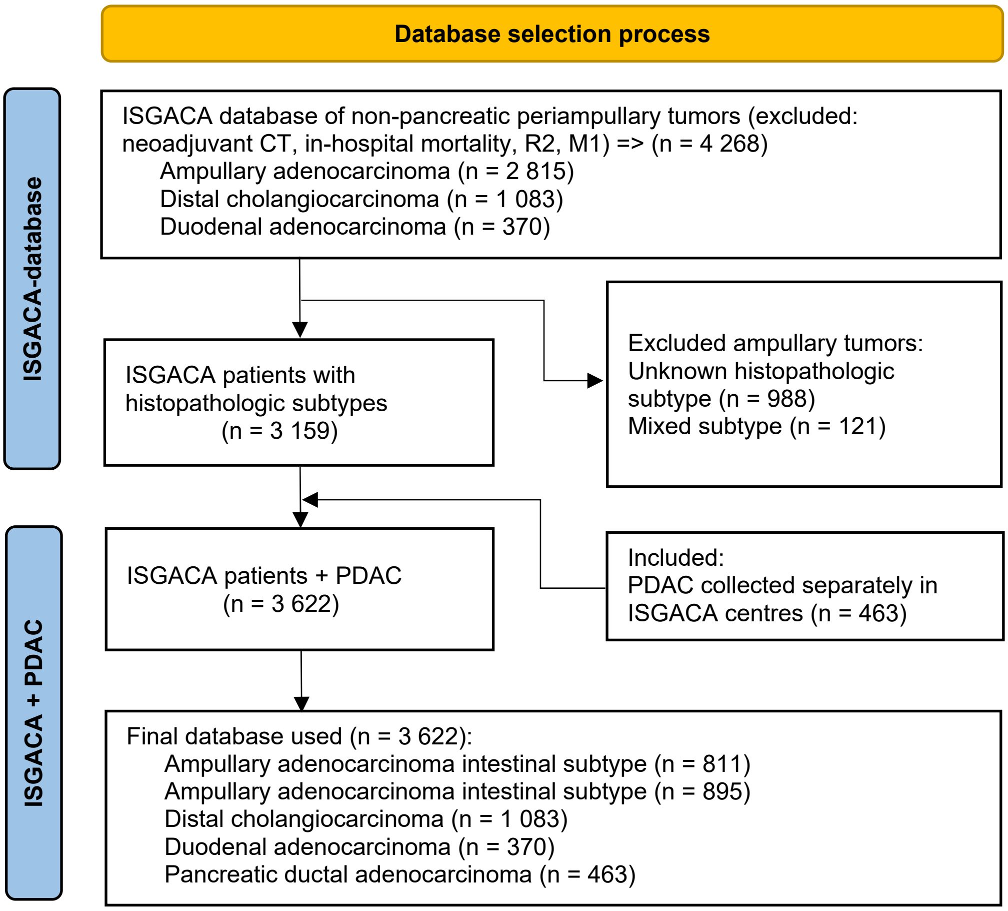

Supplementary Figure S1: Data selection process

Supp Table S1: Post-operative outcomes

	DAC	P (DAC vs AmpIT)	AmpIT	P (AmpIT vs AmpPB)	AmpPB	P (AmpPB vs dCCA)	dCCA	P (dCCA vs PDAC)	PDAC	P total
Mortality, n (%)	22 (5.9)	0.305	36 (4.3)	0.470	47 (5.2)	0.120	39 (3.7)	**0.043**	6 (1.5)	**0.013**
CR-POPF, n (%)	89 (27.3)	0.605	138 (25.5)	**0.004**	124 (18.5)	**<0.001**	211 (27.6)	**<0.001**	38 (8.3)	**<0.001**
CR-PPH, n (%)	30 (12.6)	0.086	40 (8.2)	0.371	40 (6.6)	**0.008**	70 (11.1)	0.422	3 (6.2)	**0.018**
CR-BL, n (%)	22 (6.7)	0.766	33 (6.0)	0.998	38 (5.9)	0.438	66 (7.0)	**0.018**	17 (3.7)	0.171
CR-DGE, n (%)	31 (15.1)	1.000	56 (15.3)	0.732	68 (14.3)	**0.030**	47 (9.5)	**<0.001**	6 (1.9)	**<0.001**
Hospital stay §	15 [11, 24]	**0.002**	14 [10, 21]	0.527	14 [9, 20]	**0.004**	15 [10, 24]	**<0.001**	11 [8, 17]	**<0.001**

Abbreviations: DAC, Duodenal adenocarcinoma; AmpIT, Ampullary adenocarcinoma subtype intestinal; AmpPB, Ampullary adenocarcinoma subtype pancreatobiliary; PDAC, Pancreatic ductal adenocarcinoma; P, P-value; Mortality, <30 days or in-hospital mortality; Hospital stay, Length of hospital stay in days; §, (median [IQR]); Bold values corresponds with <0.05 significance; CR-POPF, Clinically relevant postoperative pancreatic fistula ISGPS Grade B/C; CR-PPH, Clinically relevant post-pancreatectomy hemorrhage ISGPS grade B/C; CR-BL, Clinically relevant bile leakage; Length of hospital stay, reported in days.
